# Self-Efficacy to Manage Chronic Disease (SEMCD) scale: translation and evaluation of measurement properties for a swedish version

**DOI:** 10.1186/s13690-022-01022-x

**Published:** 2023-01-04

**Authors:** Jeanette Melin, Andreas Fors, Sofie Jakobsson, David Krabbe, Ida Björkman

**Affiliations:** 1 RISE Research Institutes of Sweden, Division Safety and Transport, Measurement Science and Technology, Gothenburg, Sweden; 2 Centre for Person-Centred Care (GPCC), University of Gothenburg, Gothenburg, Sweden; 3 Institute of Health and Care Sciences, The Sahlgrenska Academy, University of Gothenburg, 40530 Gothenburg, SE Sweden; 4 Region Västra Götaland, Research, Education, Development and Innovation, Primary Health Care, Gothenburg, Sweden; 5 Institute of Neuroscience and Physiology, University of Gothenburg, Gothenburg, Sweden; 6 Neurocare, Sahlgrenska University Hospital, Gothenburg, Sweden

**Keywords:** Self-efficacy, Rasch analysis

## Abstract

**Background:**

Reinforcing self-efficacy in patients is important in person-centered care; therefore, reliable and valid measures of a person’s self-efficacy is of clinical relevance. A questionnaire suitable for self-efficacy and patient engagement that is not limited to a particular condition is the Self-efficacy to Manage Chronic Disease (SEMCD). This study aims to evaluate the measurement properties of a Swedish translation of the SEMCD with a Rasch analysis.

**Methods:**

The translation and cultural adaptation of the SEMCD was performed according to the International Society for Pharmacoeconomics and Outcomes Research (ISPOR) recommendations. Self-reported data was collected from two cohorts: patients with pituitary tumors (*n* = 86) and patients on sick leave due to common mental disorders (*n* = 209). Measurement properties were evaluated with a Rasch analysis in RUMM2030.

**Results:**

The original six-item SEMCD did not fit to a unidimensional scale. Two items, item 5 and item 6, deviated both statistically and conceptually and were removed. A four-item solution, the SEMCD-4 with collapsed thresholds for mid-range response options, showed good targeting and unidimensionality, no item misfit, and a reliability of 0.83.

**Conclusion:**

In a Swedish context with a mix of patients with pituitary tumors or common mental disorders, SEMCD-4 showed satisfactory measurement properties. Thus, SEMCD-4 could be used to identify patient self-efficacy in long-term illnesses. This knowledge about patient self-efficacy may be of importance to tailor person-centered support based on each patient´s resources, needs and goals.

## Background

Prevention and management of long-term illness is a major health challenge and a global priority [1]. There is a need for future care in which each person living with a long-term illness is more involved, takes greater responsibility for and manages to a greater extent symptoms and other consequences in everyday life [2]. Person-centered care (PCC) emphasizes the importance of knowing the patient as a person, and aims to engage patients as active partners in their care and treatment [3, 4]. By acknowledging the patient’s unique experiences, capabilities, resources and needs, PCC can support a patient’s self-management and coping [3]. PCC coupled with health promotion aiming to improve self-management abilities has been suggested as a way to provide sustainable, affordable, high quality health care [5].

A concept closely related to PCC is self-efficacy, which refer to a person’s confidence in their capacity to influence events that affect their life [6]. A person’s self-efficacy beliefs have important implications for managing chronic and long-term illness [7], as patients who feel more confident in performing activities are more likely to reach treatment goals [8]. PCC seeks to reinforce self-efficacy in patients rather than to simply educate and convince them of the importance of such activities [3, 9]. There is a relationship between self-efficacy to manage chronic disease and health-related quality of life (HRQOL); patients with multi-morbidity in primary care who report higher self-efficacy also have higher HRQOL than those who report lower self-efficacy [10].

Although self-efficacy is primarily considered task-specific, meaning a person´s belief in their ability to deal with a specific situation at hand [11], it may also be perceived as a general concept, which instead refers to a person´s global confidence in their ability to perform across a variety of situations [12]. Several PCC interventions, addressing various conditions and performed in different health care contexts, have been shown to increase both illness-specific [13, 14] and general levels of self-efficacy [15, 16].

A person’s self-efficacy is typically measured by self-report questionnaires. A particularly suitable questionnaire for self-efficacy and patient engagement is the Self-Efficacy to Manage Chronic Disease (SEMCD) [17]. The SEMCD is not limited to a particular condition, rather it is intended to be useful in a broad range of situations involving individuals with varying long-term illnesses. Examples of previous applications are cardiovascular diseases, diabetes, arthritis, cancer, asthma and neurological conditions [18–24]. Furthermore, SEMCD was used in a Swedish study of graft rejection after lung transplantation [25], but without psychometric evaluation. Recently, another Swedish study translated the SEMCD and tested its psychometric properties in a population of patients with systemic sclerosis [26].

To the best of our knowledge, previous validation studies of the SEMCD have used Classical Test Theory (CTT) [17–19, 26, 27]. CTT does not adequately handle ordinal data and separates item and person attributes [28, 29]. To overcome those shortages, a measurement science perspective is needed to analyze the measurement properties of the SEMCD. According to Rasch [30], data are evaluated against a mathematical measurement model to guide the construction of stable linear measures for item and person attributes from raw data, such as questionnaire responses. By examining the extent to which observed data accord with the expected values that are defined by the measurement model, the Rasch analysis assesses if requirements for internal validity and invariant measurement across items and persons are met.

Hence, this study aims to evaluate the measurement properties of a Swedish translated version of the SEMCD with a Rasch analysis.

## Methods

### Study design and setting

The translation and cultural adaptation of the SEMCD was performed according to international recommendations [31]. The data used in this study derived from baseline assessments from two different interventional studies:

i. a single-center quasi-experimental study evaluating a person-centered practice after surgery for patients with pituitary tumors (PT) [32]; hereafter, this sample is referred to as the PT-cohort; and
ii. a randomized controlled study evaluating the effects of a person-centered eHealth intervention (combined digital platform and phone support) for patients on sick leave due to common mental disorders (CMD) [16, 33]; hereafter, this sample is referred to as the CMD-cohort.

### Participants and data collection

Table 1 summarizes the participant characteristics from the PT- and CMD-cohorts. The PT-cohort consists of 86 patients with pituitary tumors (aged 24–87) included in the intervention group between February 2018 and December 2020. All consecutive patients over the age of 18 and planned for neurosurgery due to a pituitary tumor were asked to participate. Exclusion criteria were conditions that might restrict understanding of the study or the ability to adhere to the protocol (e.g., cognitive impairments or drug addiction). Detailed information about the data collection can be found in Jakobsson et al. [32].

**Table 1:** Participant characteristics from the pituitary tumors (PT)- and common mental disorders (CMD)-cohorts and total for evaluation of the Swedish version of Self-Efficacy to Manage Chronic Disease Scale

	PT-cohort (*n* = 86)	CMD-cohort (*n* = 209)	Total (*n* = 295)
Gender			
Woman, n (%)	35 (41%)	175 (84%)	210 (71%)
Man, n (%)	51 (59%)	33 (16%)	84 (28%)
missing, n (%)		1 (0.5%)	1 (0.3%)
Age group			
≤ 35 years, n (%)	10 (12%)	75 (36%)	85 (29%)
36–45 years, n (%)	7 (8%)	52 (25%)	59 (20%)
46–55 years, n (%)	14 (16%)	50 (24%)	64 (22%)
56–65 years, n (%)	24 (28%)	31 (15%)	55 (19%)
> 65 years, n (%)	31 (36%)	1 (0.5%)	32 (11%)
Education			
Primary school, n (%)	11 (13%)	13 (6%)	24 (8%)
Secondary school, n (%)	49 (57%)	72 (34%)	121 (41%)
University, n (%)	24 (28%)	122 (58%)	146 (49%)
missing, n (%)	2 (2%)	2 (1%)	4 (1%)

The CMD-cohort consists of 209 patients (aged 18–65) on sick leave due to common mental disorders (stress and adjustment disorders, depression, or anxiety) who were recruited between February 2018 and June 2020 from primary care centers and randomly assigned to the intervention or control group. Patients were eligible if they were currently employed or studying at least part-time during the past nine months, and if their current sick leave period had not exceeded 30 days. Exclusion criteria were: sick leave > 14 days due to depression, anxiety disorders, stress reactions and disorders during the past 3 months; severe impairments impeding use of the eHealth intervention; ongoing alcohol or drug abuse; severe disease with an expected survival of < 12 months or that could interfere with follow-up or if the intervention was assessed as a burden; or the patient was participating in a conflicting study. Details about the data collection can be found in Cederberg et al. [16, 33].

### Measurements

The SEMCD is a questionnaire developed for the assessment of a patient’s confidence in their ability in certain tasks related to self-management (see Table 3 in the Results section for items). With the SEMCD the patient is asked to rate his or her confidence related to six tasks on a 1 to 10 scale (1 anchored to “not at all confident” and 10 to “totally confident”) [17].

The SEMCD was developed in the U.S. by Lorig and co-workers [17, 21] and has been translated into Spanish [21, 24], German [18], French [34], Chinese [35, 36], Turkish [19], Portuguese [37], Persian [38], Arabic [39] and Swedish [26]. A comparison of the translations in the other Swedish study and the present translation shows that the content is the same, but the word-order is different.

In this study, the recommendations from the International Society for Pharmacoeconomics and Outcomes Research (ISPOR) [31] were followed to translate and adapt the SEMCD. The process is shown in Table 2.

**Table 2:** Translation and adaptation process for the Swedish version of Self-Efficacy to Manage Chronic Disease (SEMCD) Scale

Step		Description for SEMCD
1	Preparations	The research group planned for the translation process and permission to use the SEMCD was obtained from its developer, professor K Lorig in March 2017
2	Forward translation	Two independent professional translators carried out translations from English to Swedish
3	Reconciliation	The research group compared the two translations and reconciled them via discussion. There were some minor differences, which could be solved within the research group
4	Backward translation	Two new independent translators, one a professional translator and one a native English-speaking researcher, carried out backward translation from Swedish to English
5	Backward translation review	The research group reviewed the backward translations to ensure the conceptual equivalence of the translation of the Swedish version of the SEMCD
6	Harmonization	After the review of the backward translation, the research group concluded that there was a harmonized translation
7	Cognitive debriefing	The translated Swedish version of the SEMCD was tested with six respondents to assess the comprehensibility and conceptual understanding of the SEMCD. The respondents were all native Swedish speakers from the PT-cohort
8	Reviews of the cognitive debriefing results and finalization	The cognitive debriefing did not lead to any changes
9	Proof-reading	All members in the research group proofread the Swedish version of the SEMCD before it was dispatched to the patients included in this study
10	Final report	This paper is the final report of the translation process carried out for the translation of the SEMCD into Swedish

The General Self-Efficacy Scale (GSE) is a questionnaire for the assessment of a patient’s confidence in their ability to cope with and adapt to difficult problems and challenges [40]. With the GSE the patient is asked to rate his or her confidence for ten challenges on a four-point scale (1 = “not at all true”; 2 = “hardly true”; 3 = “moderately true”; and 4 = “exactly true”). The GSE was originally developed in Germany and has been translated to and used in more than 30 languages, including Swedish, and is broadly used internationally [12].

### Data analysis

The item and person attributes are so-called coupling attributes [41]. In this study the questionnaire item attribute measures range from easy to difficult tasks to manage, hereafter referred to as *item difficulty measures.* The person attribute measures range from lower to higher confidence in self-management ability, hereafter referred to as *self-efficacy measures*. An easy task to manage corresponds to an item with high probability of success while a difficult task to manage corresponds to an item with low probability of success. Consequently, a person with a lower self-efficacy score has a lower probability of success with self-efficacy tasks that are difficult to manage.

For all analyses the software *Rasch Unidimensional Measurement Model 2030* (RUMM2030) was used and a partial credit model was applied, i.e., each item was allowed to have its own rating scale structure. Correlations were done in SPSS version 27. The Rasch analysis was structured around the fundamental measurement aspects of the Rasch model [29, 42].

#### Targeting of persons and items

Comparing mean person location with mean item location (i.e., 0 logits) indicates whether or not the sample is off-centered with respect to the items [42]. Moreover, person fit residuals should ideally lie within the -2.5 to + 2.5 range [29]. Together with the fit statistics, examining how the items are distributed along the continuum is crucial for deciding whether a measurement scale can successfully be constructed or not [29]. This means that one must consider if the item distribution is consistent with clinical or theoretical expectations [43].

#### Threshold ordering

To evaluate the monotonicity of items the threshold orders were evaluated; the ratings on one item should be consistent with the metric estimate of the underlying construct. Collapsing categories was considered when disordered thresholds occurred [42].

#### Item model fit

Items were assessed according to fit residuals, chi-squared tests and the Item Characteristic Curve (ICC). The following guidelines were applied iteratively: i) mean fit residuals should be close to zero (0) and have standard deviations (SD) close to 1; ii) the individual item fit residuals should be between -2.50 and + 2.50; iii) the chi-squared values should not be statistically significant (Bonferroni corrected); and iv) the dots of the class intervals should follow the ICC to support good fit [29].

#### Differential Item Functioning (DIF)

For DIF analysis both main effects and interaction effects were taken into account; both were expected to be non-significant. Because of the multiple tests, Bonferroni correction was applied. DIF was tested for cohort, gender, age group and education accordingly (see participant characteristics in Table 1). When DIF was observed, item splits were evaluated in repeated analyses starting with the item with largest DIF [44].

#### Local dependency

To evaluate local dependency among items, residual correlations were compared against a relative cut off; residual correlations > 0.20 above the average correlations indicated local dependency [45].

#### Unidimensionality

Having items in a questionnaire measure only a single construct is a fundamental requirement for the Rasch model; unidimensionality should therefore be evaluated. Smith’s [46] method for testing unidimensional is recommended. First, the patterning of residuals is evaluated in a Principal Component Analysis (PCA); second, the first residual factor is used to define two subsets of items by dividing positively and negatively correlated items; and third, the person estimates for each subset are compared by using an independent t-test. To support unidimensionality, the percentage of tests outside the range -1.96 to 1.96 should not exceed 5%.

#### Reliability

To evaluate the internal consistency reliability, the Person Separation Index (PSI) was used. The PSI is equivalent to Cronbach’s alpha and interpreted as follows: zero (0) indicates all errors/noices and one (1) no error.

#### Group differences

ANOVA was used to evaluate group differences for cohort, gender, age group and education [29].

In addition, correlation between self-efficacy measures from SEMCD and GSE was evaluated. We expected the correlation to be positive but not strong as the SEMCD and GSE assesses different dimensions of self-efficacy. Assessment of the measurement properties of GSE is beyond this study. However, to perform statistical analyses (both creating a total “GSE measure” and correlations) we applied the Rasch formula to derive linear GSE person measures. In short, the GSE did not have any misfitting items, response options worked as intended, and although the PSI was > 0.90 it was slightly positively skewed (mean location 0.683; SD 2.063).

## Results

The results are presented in four sections. First results from the Rasch analysis of the full scale SEMCD are reported. Second, rationales and suggestions for modifications are presented. Third, results from the Rasch analysis of the modified SEMCD are reported. Finally, we report the correlations between the modified SEMCD and the GSE.

### Initial analysis – SEMCD full scale

#### Targeting of persons and items

Mean self-efficacy measures were close to zero (-0.0049) indicating a well targeting between the sample and the items. Furthermore, 5 (1.7%) persons had fit residuals > 2.5 and 18 (6.1%) persons had fit residuals < 2.5.

#### Threshold ordering

Disordered thresholds were present for item 1 [*Confidence in keeping fatigue from interfering*] and item 3 [*Confidence in keeping emotional distress from interfering*]. For the remaining items thresholds were ordered but narrow in the middle of the response options.

#### Item model fit

Mean fit residuals were close to zero (0.075), but the SD was larger than desired (2.874). All items except item 6 [*Confidence in reduce the illness effects*] showed fit residuals within the desired range of ± 2.5 and non-significant chi-square values (item 6 chi-square 5.798, *p* < 0.001). Table 3 summarizes all fit statistics.

**Table 3:** Summary of fit statistics for the Swedish version of the Self-Efficacy to Manage Chronic Disease (SEMCD) scale showing full scale and modified versions. The SEMCD item 1-4 where response option 4-5-6 is collapsed for item 1 is further reported in the section on SEMCD-4.

								Differential item functioning (DIF)			
	Item number	Item	Location	2SE	FitResid	Prob	Disordered thresholds	Cohort	Gender	Age Group	Education
SEMCD—full scale	1	Confidence in keeping fatigue from interfering	0.36	0.07	-0.91	0.02	x	x		x	
	2	Confidence in keeping pain/physical discomfort from interfering	-0.06	0.06	-0.35	0.01					
	3	Confidence in keeping emotional distress from interfering	0.30	0.07	-1.31	0.06	x	x	x	x	
	4	Confidence in keeping other symptoms from interfering	0.02	0.07	-2.16	0.01					
	5	Confidence in reduce need to the doctor	-0.13	0.07	-0.63	0.45					
	6	Confidence in reduce the illness effects	-0.49	0.06	**5.80**	**0.00**		x	x	x	x
SEMCD item 1–5	1	Confidence in keeping fatigue from interfering	0.30	0.08	-0.69	0.11	x		x		
	2	Confidence in keeping pain/physical discomfort from interfering	-0.18	0.07	0.22	0.09					
	3	Confidence in keeping emotional distress from interfering	0.26	0.08	-1.31	0.23		x			
	4	Confidence in keeping other symptoms from interfering	-0.10	0.07	-1.22	0.10					
	5	Confidence in reduce need to the doctor	-0.28	0.07	**3.06**	0.32					
SEMCD item 1–4	1	Confidence in keeping fatigue from interfering	0.25	0.08	-0.31	0.13	x				
	2	Confidence in keeping pain/physical discomfort from interfering	-0.28	0.08	-0.10	0.10					
	3	Confidence in keeping emotional distress from interfering	0.22	0.08	-0.72	0.22		x	x		
	4	Confidence in keeping other symptoms from interfering	-0.19	0.08	0.99	0.49					
SEMCD item 1–4, response options 4–5-6 collapsed for item 1	1	Confidence in keeping fatigue from interfering	0.47	0.10	0.44	0.03				x	
	2	Confidence in keeping pain/physical discomfort from interfering	-0.36	0.08	-0.10	0.07					
	3	Confidence in keeping emotional distress from interfering	0.16	0.08	-0.48	0.56		x	x		
	4	Confidence in keeping other symptoms from interfering	-0.27	0.08	1.23	0.16					

#### DIF

Item 6 [*Confidence in reduce the illness effects*] showed significant DIF for all comparisons (cohort, gender, age group and education). Item 3 [*Confidence in keeping emotional distress from interfering*] showed significant DIF for cohort, gender and age group comparisons. Item 1 [*Confidence in keeping fatigue from interfering*] showed significant DIF for comparisons between cohorts and age groups.

#### Local dependency

The average residual correlation was -0.173, giving a relative cut-off of 0.027. Three residual correlations were above the cut-off: item 1 [*Confidence in keeping fatigue from interfering*] and item 2 [*Confidence in keeping pain/physical discomfort from interfering*] (0.090); item 1 [*Confidence in keeping fatigue from interfering*] and item 3 [*Confidence in keeping emotional distress from interfering*] (0.175); and item 5 [*Confidence in reduce need to the doctor*] and item 6 [*Confidence in reduce the illness effects*] (0.099).

#### Unidimensionality

The positively loaded item residuals in the first residual factor were item 5 [*Confidence in reduce need to the doctor*] and 6 [*Confidence in reduce the illness effects*]. The remaining four item residuals were negatively loaded. The comparison of the person self-efficacy measures from those subsets, i.e., items 1–4 and items 5–6, had 7.85% persons outside the desired range of ± 1.96.

#### Reliability

The PSI without extremes was 0.834 and with extremes 0.862.

#### Group differences

There was no statistically significant difference in self-efficacy measures based on education (p = 0.243). For the other group comparisons: the PT-cohort showed higher self-efficacy compared to the CMD-cohort (*p* < 0001), older (> 65 years) showed higher self-efficacy compared to younger (< 35 years) (*p* < 0001), and men showed higher self-efficacy compared to women (*p* < 0001).

### Rationale for modification of the SEMCD

Item 6 [*Confidence in reduce the illness effects*] had several drawbacks in terms of individual item fit and effect on unidimensionality. It is also conceptually different from all other items in the SEMCD. As described previously [17], there is a difference between items 5 and 6 compared to items 1–4 in terms of area of confidence assessed. Items 5 and 6 concern confidence to do things to manage the disease, whereas items 1–4 concern confidence to keep aspects of the disease from interfering with things a person wants to do [17]. When removing item 6, item 5 [*Confidence in reduce need to the doctor*] showed minor misfit. Due to its conceptual difference from items 1–4 as well as its somewhat misleadingly referring only to the doctor and no other profession, item 5 was also removed. This action corresponds well with the suggested argument for changing ‘see a doctor’ to ‘meet health care’ suggested by Mattsson et al. [26]. Further, we iteratively assessed response category function and DIF. After these iterative analyses the optimum result was obtained when removing both item 5 and item 6 as well as collapsing the middle (4–5-6) response options for item 1 [*Confidence in keeping fatigue from interfering*], giving SEMCD-4.

### SEMCD-4

#### Targeting of persons and items

**Fig. 1:**
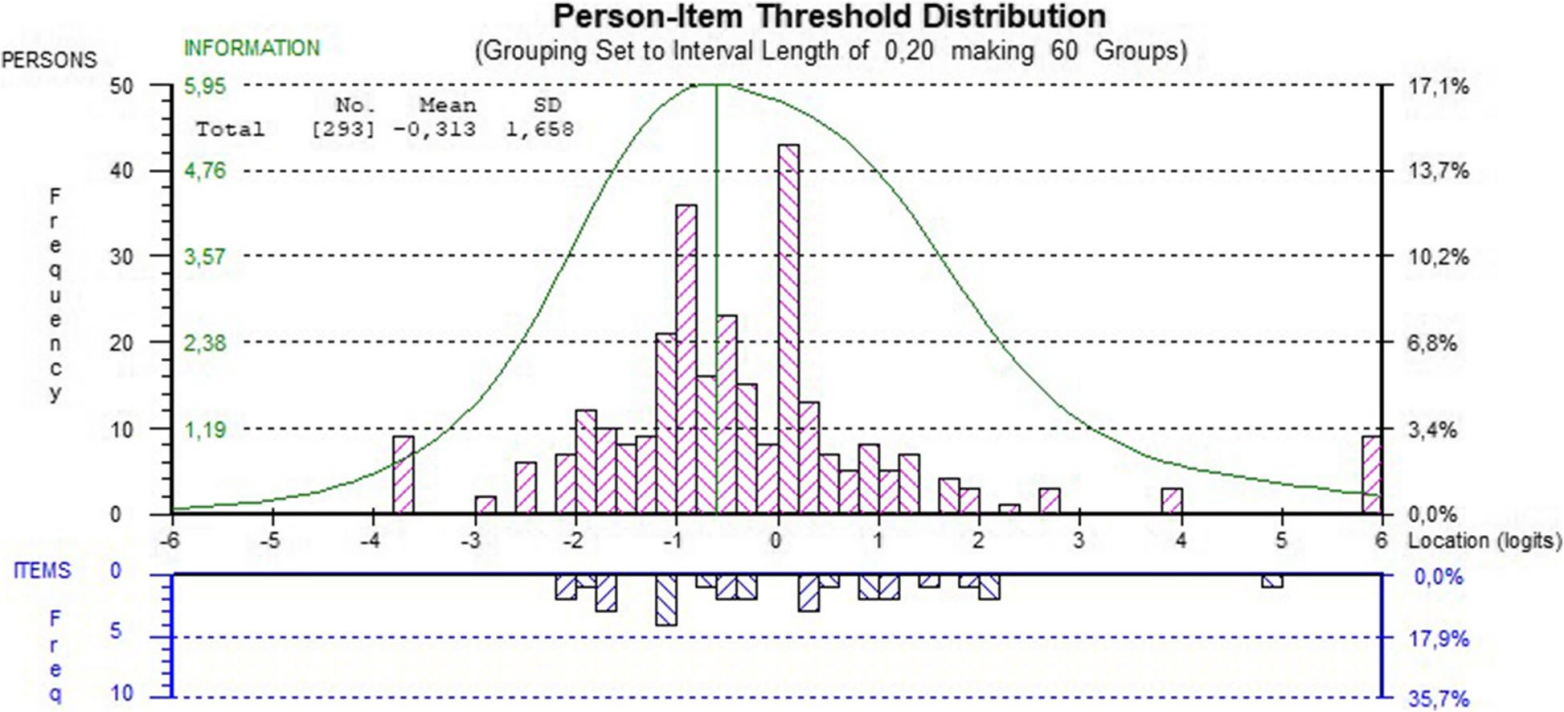
Person-Item threshold distributions for the swedish version of Self-Efficacy to Manage Chronic Disease (SEMCD) scale. Top histogram (pink bars) showing sample distribution of person self-efficacy measures and bottom histogram (blue bars) distribution of location of item thresholds in the SEMCD-4. Green line shows information curve

Mean person location was -0.294 (SD 1.592), indicating good sample-item targeting. However, as shown in the person-item threshold histogram in Fig. 1, item thresholds are not covered by the person thresholds. Specifically, there is a gap in item thresholds between 1.936 and 4.844 logits, where the higher threshold – alone – belongs to item 1 [*Confidence in keeping fatigue from interfering*].

In total, 1 (0.3%) person had fit residual > 2.5, i.e., an unexpected irregular response pattern, and 19 (6.5%) persons had fit residuals < 2.5, i.e., low variations in their response patterns.

#### Threshold ordering

As response categories were collapsed to resolve disordered thresholds, all thresholds became ordered.

#### Item model fit

Mean fit residuals were 0.501 (SD 0.765) and item fit residuals were within the desired range of ± 2.5 with non-significant chi-square values (Table 3), supporting unidimensionality.

We did not have any a priori understanding of easier or more difficult items (i.e., the probability of success), but the analyses showed that item 2 [*Confidence in keeping pain/physical discomfort from interfering*] was the easiest, followed by item 4 [*Confidence in keeping other symptoms from interfering*], then item 3 [*Confidence in keeping emotional distress from interfering*] and then item 1 [*Confidence in keeping fatigue from interfering*] (location column in Table 3).

#### DIF

**Fig. 2:**
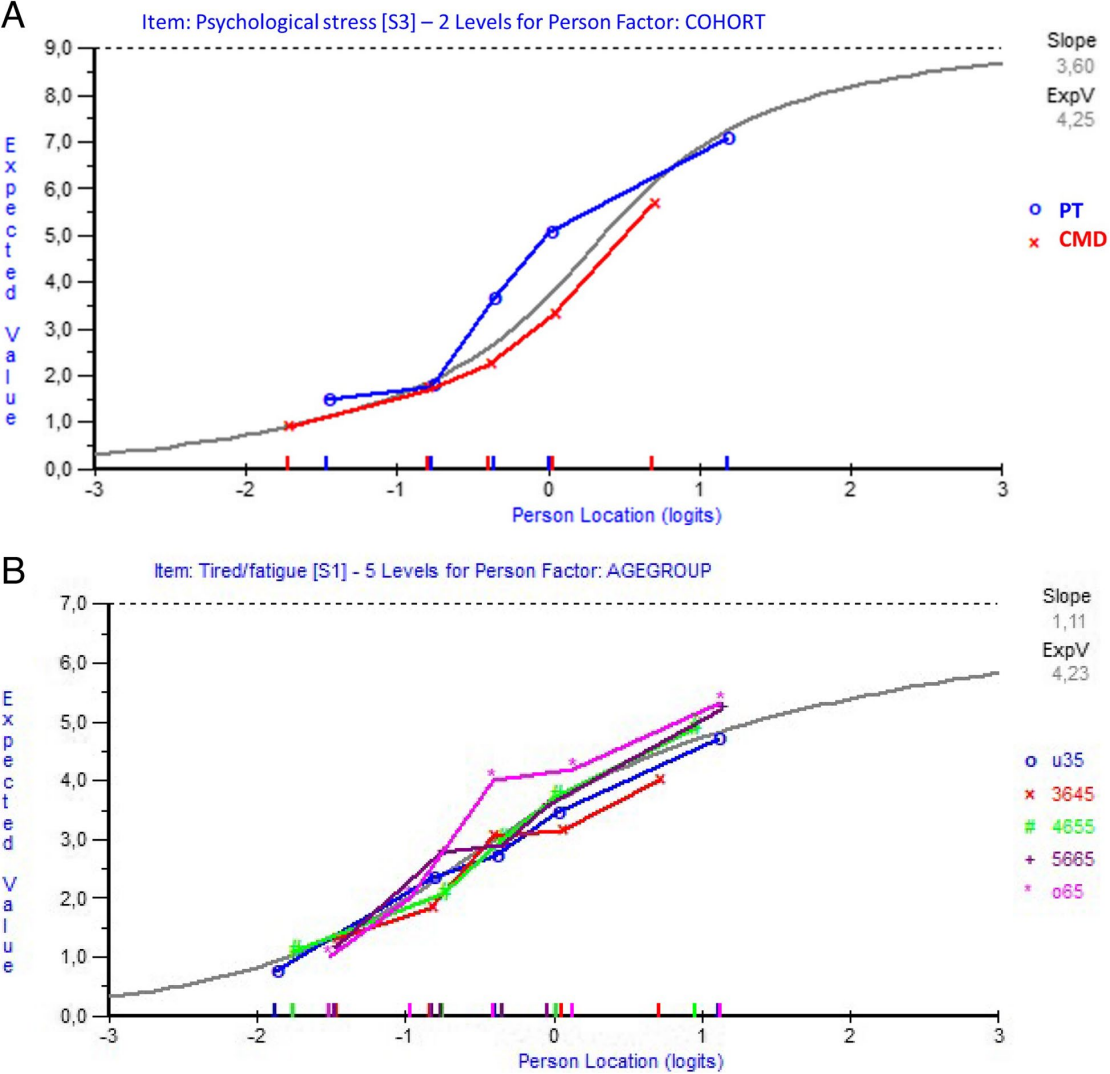
**a**-**b** Differential item functioning curves for the swedish version of Self-efficacy to Manage Chronic Disease scale. Differential item functioning curves for **a** item 3 [Confidence in keeping emotional distress from interfering] for the cohort groups (pituitary tumors-cohort in blue and common mental disorders-cohort in red) and **b** item 1 [Confidence in keeping fatigue from interfering] for the different age groups

Item 3 [*Confidence in keeping emotional distress from interfering*] showed significant DIF between the cohorts (Fig. 2a) as well as for gender. Item 1 [*Confidence in keeping fatigue from interfering*] showed significant DIF for comparisons between age groups (Fig. 2b). The DIF on item 3 was successfully resolved by splitting the item for cohort, but not gender. The item split showed that for item 3 there were approximately 4 logits difference between the cohorts, where it was easier for the PT-cohort compared to the CMD-cohort (i.e., the PT-cohort had a higher probability of success managing emotional stress). The DIF on item 1 was successfully resolved by splitting the item for the oldest cohort members (over 65 years); the older cohort members endorsed item 1 1.2 logits higher. However, splitting items did not change the person self-efficacy measures significantly.

#### Local dependency

The average residual correlation was -0.317, giving the relative cut-off of -0.117. The item residual correlation between item 1 [*Confidence in keeping fatigue from interfering*] and item 3 [*Confidence in keeping emotional distress from interfering*] was slightly above the average cut-off (-0.083).

#### Unidimensionality

Item 1[*Confidence in keeping fatigue from interfering*] and item 2 [*Confidence in keeping pain/physical discomfort from interfering*] had positively correlated item residuals in the first residual factor, and item 3 [*Confidence in keeping emotional distress from interfering*] and item 4 [*Confidence in keeping other symptoms from interfering*] were negatively correlated. When comparing the person estimates from the subsets, less than 5% (4.44%), were outside the desired range of ± 1.96. These findings support unidimensionality.

#### Reliability

Despite removing items as well as collapsing response options, the high reliability was not affected. The PSI without extremes was 0.829 and with extremes 0.868.

#### Group differences

The same statistically significant and non-significant differences as for the full scale were shown. These were: no difference for education (*p* = 0.129); a difference in which the PT-cohort showed higher self-efficacy compared to the CMD-cohort (*p* < 0001); older (> 65 years) showed higher self-efficacy compared to younger (< 35 years) (*p* < 0001); and men showed higher self-efficacy compared to women (*p* < 0001).

### Correlation between SEMCD-4 and GSE

As expected, there was a positive correlation between the person self-efficacy measures from SEMCD-4 and GSE (Pearson correlation coefficient 0.424, *p* < 0.001). For subgroups, the lowest correlation was observed among 36–45-year-old persons (Pearson correlation coefficient 0.155; *p* = 0,243) and the highest correlation was observed among 56–65-year-old persons (Pearson correlation coefficient 0.537; *p* < 0.001).

## Discussion

This article reports the translation of the SEMCD and an evaluation of its measurement properties. Our findings propose that a four-item solution, SEMCD-4, fits well to the measurement model in a Swedish context, which implies satisfactory measurement properties.

Removing items has, at least, two critical implications. First, the question arises as to whether parts of the initial item set could be used to measure the same attribute or if only one aspect of the attribute is being measured. Previous work has shown difficulties fitting the six items together into one dimension [17], and the results in this study confirm this difficulty. Recently, the PROMIS®-network has provided an item bank for measuring self-efficacy in chronic conditions [47]. Based on a Delphi panel they have divided the construct and proposed five different dimensions of self-efficacy management: daily activities, medications and treatment, symptoms, emotions, and social interactions. The symptom dimension showed a strong correlation (r = 0.76) with the SEMCD, which is not surprising as it comprises four specific symptom items [47]. The major difference between our proposed SEMCD-4 and the PROMIS® symptom dimension item bank is that SEMCD-4 measures four general symptom management items compared to the PROMIS® item bank with 28 items addressing confidence in symptom management, symptom management in different settings, and in keeping symptoms from interfering with daily activities [47]. Thus, taking previous work and the results of this study into consideration, the unidimensionality of the construct self-efficacy to manage chronic disease is questionable. However, were there greater equality in the number of items covering the different aspects of self-management (there is a need to add items to SEMCD), it might be possible to create a higher ordered measure of self-efficacy for self-management in long-term illnesses. This hypothesis, however, needs further investigation. A second critical implication is that comparability might be lost in removing items. From the perspective of the CTT, this conjecture is correct. The Rasch model, applied in this study, offers a logical progression from the CTT paradigm to a more sophisticated model [48], namely, a specific metrological approach to human-based measurements [41], to overcome the shortages with ordinality and lack of separation between item and person attributes. A key factor for comparability, independent of which items are included, is traceability to metrological references, which is enabled with calibrated items [41, 49, 50]. Thus, applying the Rasch model ensures improved possibilities for metrological comparability. Specifically, sets of items (e.g., SEMCD and SEMCD-4) can be linked to a common self-efficacy measures by means of co-calibration of item parameters [50].

With regards to the item hierarchy of SEMCD-4, it was evident that is it easier being confident managing physical symptoms than symptoms dominated by psychological components. That is, there is a higher probability of scoring higher on the item for managing physical symptoms or pain compared to the items for managing emotional stress or fatigue. This difference may be due to the symptomatology of the cohorts. Symptoms of fatigue and being more sensitive to stressful situations are prevalent in CMD [51, 52], and fatigue is found to be among the most frequent and bothersome complaints reported by patients treated for pituitary disease [53, 54]. Another explanation could be that physical symptoms are more closely related to clinical signs (e.g., feeling hot or flushed, body aches, and weakness are symptoms of fever), which are still often emphasized in clinical assessments [55] and thereby presumably given more attention and intervention. However, that possible explanation is undercut by the fact that medically unexplained physical symptoms and pain are health problems that are increasing in the western world [56]. We would therefore encourage further investigation into the item hierarchy and the relation between different aspects of self-efficacy in different clinical settings.

Because of the need for self-report questionnaires to evaluate self-efficacy in managing chronic diseases, two Swedish versions of the SEMCD have been developed in parallel: the SEMCD-4 evaluated in this present study and the recently investigated SEMCD-Swe [26]. As mentioned above, the SEMCD-Swe and our work show comparable content but different word-order, which might not be a major issue. Furthermore, respondents in the Mattsson et al. [26] study noted that items 5 [*Confidence in reduce need to the doctor*] and 6 [*Confidence in reduce the illness effects*] were difficult to understand. They had to allow for covariance between those items to fit a single-factor structure, but choose to keep them in their final version. Different length questionnaires can be confusing, but the benefit of the Rasch analysis in this study is that is showed that only the four symptom items can be combined with validity, something that is supported by previous work [17].

In this cross-sectional study, we showed positive moderate correlations between person measures from the SEMCD and GSE. This corroborates other studies that evaluated correlations between GSE and specific self-efficacy scales [12, 18, 57]. Considering that both specific self-efficacy scales and the GSE originate from the concept of self-efficacy, such positive correlations are expected. However, the correlation between the SEMCD and GSE was not strong enough to consider them to be measuring the same thing. Hence, it is possible to exhibit higher general self-efficacy and at the same time lower self-efficacy to manage a chronic disease, and vice versa. However, their relationship over time after an intervention of person-centered care is unexplored.

The selection of the two cohorts in this study was based on their clinical differences (e.g., etiology, symptom expression, treatment, different care levels) but also on their similarity in both being long-term illnesses for which self-management is a major part of care. In line with the original idea of the SEMCD, it should not be restricted to a specific diagnosis or setting but rather used to target self-efficacy for self-management in general [17]. At this stage, however, generalizability of our results to other long-term illnesses is limited. There was also a practical reason for the choice of cohorts as in both cases the interventions are evaluating the effects of PCC and both use self-efficacy as an outcome measure. The studies were not specifically designed to assess the measurement properties of SEMCD. Shortcomings include not having appropriate sample sizes and not equally balanced cohorts in terms of diagnostic group. However, the possibility of including SEMCD in intervention studies in the future presents opportunities for further analysis of sensitivity to change and item stability over time.

This study has implications for clinical practice given the global burden of long-term and chronic illness and the concomitant need for person-centered interventions supporting patients’ self-management. The SEMCD-4 is well suited to identify patients with lower levels of self-efficacy and thus probably in higher need of support to manage symptoms in long-term illness, with that support tailored to individual needs. The SEMCD-4 also has a low respondent burden and worked well in our two cohorts; it can therefore possibly be used in research and clinical practice to evaluate interventions aiming at increased self-management in different patient groups and clinical settings.

## Conclusion

In a Swedish context with patients with pituitary tumors or common mental disorders, the SEMCD-4, a four-item solution that includes management of symptoms, shows satisfactory measurement properties. For the clinical groups studied here, and perhaps beyond, the SEMCD-4 could be used to identify patient self-efficacy for managing long-term illness. It could also be used to tailor person-centered support based on each patient´s resources, needs and goals.

We encourage further work to explore a higher ordered measure of self-efficacy for self-management in long-term illness. A higher-ordered measure is warranted when including more self-efficacy related activities beyond symptom management, requiring investigations of the item hierarchy and relations between different self-efficacy items across different clinical settings.

## Data Availability

The datasets used during the current study are available from the corresponding author on reasonable request.

## Abbreviations

CMD: Common mental disorders
CTT: Classical Test Theory
DIF: Differential Item Functioning
GSE: General Self-Efficacy Scale
HRQOL: Health-related quality of life
ICC: Item Characteristic Curve
ISPOR: International Society for Pharmacoeconomics and Outcomes Research
PCA: Principal Component Analysis
PCC: Person-Centered Care
PSI: Person Separation Index
PT: Pituitary tumors
SD: Standard deviations
SEMCD: Self-efficacy to Manage Chronic Disease
